# RETRACTION: lncRNA SNHG14 Plays a Role in Sepsis-Induced Acute Kidney Injury by Regulating miR-93

**DOI:** 10.1155/mi/9875328

**Published:** 2025-07-21

**Authors:** Mediators of Inflammation

RETRACTION: C. Shi, Y. Zhao, Q. Li and J. Li, “lncRNA SNHG14 Plays a Role in Sepsis-Induced Acute Kidney Injury by Regulating miR-93,” *Mediators of Inflammation* 2021, no. 1 (2021): 1–10, https://doi.org/10.1155/2021/5318369.

The above article, published online on 06 January 2021 in Wiley Online Library (wileyonlinelibrary.com), has been retracted by agreement between the journal Editor-in-Chief, Anshu Agrawal, and John Wiley & Sons Ltd. The retraction has been agreed upon after concerns were raised by a third party regarding the use of an incorrect primer for the SNHG14 gene. Following an investigation, evidence of image manipulation was also identified in the IRKA4 row of the western blot presented in Figure 4d. The authors were invited to provide comments and supporting data but did not respond. Given the nature of the concerns, the editors consider the results and conclusions of this article invalid.

